# Common CYP2D6, CYP2C9, and CYP2C19 Gene Variants, Health Anxiety, and Neuroticism Are Not Associated With Self-Reported Antidepressant Side Effects

**DOI:** 10.3389/fgene.2019.01199

**Published:** 2019-11-26

**Authors:** Simran Maggo, Martin A. Kennedy, Zoe A. Barczyk, Allison L. Miller, Julia J. Rucklidge, Roger T. Mulder, James A. Foulds

**Affiliations:** ^1^Department of Pathology and Biomedical Science, University of Otago, Christchurch, New Zealand; ^2^Department of Psychological Medicine, University of Otago, Christchurch, New Zealand; ^3^Department of Psychology, University of Canterbury, Christchurch, New Zealand

**Keywords:** pharmacogenetics, adverse reactions, social media advertising, personalized medicine, Psychiatry

## Abstract

Many patients prescribed an antidepressant stop taking it because of side effects. Genetic factors and psychological factors including state or trait anxiety, may explain variation in side effect outcomes. Our aim was to examine the relative contribution of genetic and psychological factors in people with self-reported antidepressant side effects. We undertook a case control study (n = 194) of people who took a selective serotonin reuptake inhibitor (SSRI) or serotonin/noradrenaline reuptake inhibitor (SNRI) in the past 2 years, recruited *via* social media advertising. Cases had previously not tolerated at least one trial of an SSRI or SNRI, evidenced by stopping the drug or reducing the dose by at least 50% because of a side effect. Control participants had taken an SSRI or SNRI but did not meet case criteria. Variation in the genes *CYP2D6*, *CYP2C19*, and *CYP2C9* was analyzed by Sanger sequencing on DNA extracted from blood or saliva. Participants completed the Short Health Anxiety Inventory—18, K10, and NEO-FFI-3 personality questionnaire. Participants were 87.1% female. 70.8% had a current K10 score of 22 or more. There was no consistent evidence that cases had higher psychological distress, health anxiety, or neuroticism. There was low correspondence between participants’ CYP2D6, CYP2C19, and CYP2C9 phenotypes and their history of antidepressant tolerability. For this cohort of patients a history of not tolerating SSRI or SNRI therapy was not associated with variation in the pharmacogenes we tested, nor was it associated with health anxiety or neuroticism.

## Introduction

Antidepressants are used to treat many mental health conditions, but they have only modest efficacy in most situations (Hidalgo et al., 2007; Fournier et al., 2010; Mitchell et al., 2013) and their use is limited by side effects (Papakostas, 2008). One way to improve tolerability and therefore effectiveness may be through personalized drug prescribing. This involves using individual genetic data to inform drug and dose selection (Singh, 2015; Bousman and Hopwood, 2016 Wishart, 2016). Over time, this technology has become much cheaper and more widely available. Genetic testing products are now increasingly being marketed direct to consumers, despite some ethical concerns about this practice (European Society of Human Genetics, 2010).

Most of the advancement in antidepressant drug therapy in the past 50 years has been in tolerability, not efficacy. However, the drug classes now in most common use, the selective serotonin reuptake inhibitors (SSRIs), and serotonin and noradrenaline reuptake inhibitors (SNRIs), are still not free of tolerability problems (Carvalho et al., 2016). Some produce unpleasant withdrawal syndromes (Fava et al., 2015; Fava et al., 2018) while most are associated with side effects at higher doses, including sweating, sexual dysfunction, and in some cases serotonin toxicity (Bet et al., 2013). Cardiovascular, hepatotoxic, and metabolic effects are further potential issues (Carvalho et al., 2016).

The SSRIs and SNRIs are all metabolized by cytochrome P450 (CYP) enzymes in the liver, particularly CYP2D6, CYP2C19, and CYP2C9. The relative contribution of each enzyme varies between drugs. Genetic variation in these CYP enzymes produces individual variation in drug clearance (Tracy et al., 2016). In some ethnic groups, reduced activity in one or more CYP enzymes is common (Hicks et al., 2015). For example, about 5–7% of Caucasians are CYP2D6 poor metabolizers (PMs) (Tamminga et al., 2001), suggesting they may be less likely to tolerate drugs metabolized by CYP2D6 such as the SSRI fluoxetine.

Genetically informed prescribing guidelines are now available, such as those from the Clinical Pharmacogenetics Implementation Consortium (CPIC; www.cpicpgx.org). These guidelines help prescribers choose the “optimal” antidepressant and its starting dose for patients with known CYP phenotypes (Hicks et al., 2015). However, while pharmacogenetic testing is becoming more common, uptake has been low even among psychiatrists, suggesting clinicians either have not yet seen the value of this testing, or cannot easily access it (Foulds et al., 2016).

Patients who have already had problems tolerating an antidepressant are an important group because they are at risk of poorer overall clinical outcomes (Papakostas, 2008; Maggo et al., 2019). While it seems logical that CYP variants involving reduced drug clearance would be over-represented in this group, it is not well established that this is actually the case. Furthermore, other factors such as patient expectations, prior conditioning, anxiety, and somatization also influence drug tolerability (Barsky et al., 2002), potentially masking any effect of CYP status.

On this background, we tested two hypotheses regarding patients with a history of difficulty tolerating treatment with an SSRI or SNRI:

1. That compared to people who have tolerated antidepressant therapy, those with side effects are more likely to have genetic variants that adversely affect function of the drug metabolizing enzymes CYP2D6, CYP2C19, or CYP2C9.
2. That markers of state or trait anxiety (high scores on measures of neuroticism, health anxiety or psychological distress) are over-represented among people who have difficulty tolerating drug treatment.

## Methods

### Design

A case-control design was used. The New Zealand Northern B Health and Disability Ethics Committee (18/NTB/21) approved the study. Reporting was intended to conform to STROBE guidelines (von Elm et al., 2007).

Cases were those participants who reported any lifetime history of “*not tolerating*” any SSRI or SNRI, which was defined as having taken at least 1 dose and subsequently having discontinued the drug or reduced its dose by at least 50% (while taking a dose less than or equal to the published maximum dose) because of a drug side effect.

Control participants were those who had a history of SSRI or SNRI exposure but had no such history of tolerability problems. This definition of “caseness” was chosen because SSRIs and SNRIs have a broad range of possible side effects, and it would have been difficult to define case inclusion criteria and severity thresholds based on all possible side effects.

### Setting and Participants

A “convenient” sample was obtained *via* social media advertising on Facebook using two strategies:

1. Targeted information about the study was made available to a group of primarily New Zealand-based social media users with an existing connection to a Facebook group with an interest in mental health.
2. Social media advertising on Facebook using keywords related to depression and antidepressant therapy.

To be included in the study, participants needed to fulfill the following criteria:

1. Capacity to consent to providing a saliva or blood sample for pharmacogenetic testing
2. Age 16 or over
3. Resident in New Zealand
4. Taken at least one dose of a selective serotonin reuptake inhibitor (SSRI) and/or selective serotonin and noradrenaline reuptake inhibitor (SNRI) within the past 2 years
5. Able to complete personal health and psychometric assessment questionnaires online or on a computer.

### Drug Response Information

Participants were asked about their current and lifetime history of antidepressant use, including the names and doses of all antidepressant drugs previously taken, the duration of each treatment trial, and why any drugs were stopped or reduced in dose. For those who had previously stopped a drug or reduced the dose, the reason for this was recorded as being either primarily because of a drug side effect or for other reasons (including lack of efficacy). To minimize respondent burden, participants were asked to describe the side effect(s) leading to discontinuation in their own words. Where information provided by participants was unclear or incomplete, participants’ electronic medical records were consulted.

### Genotyping and Phenotyping

Genomic DNA was extracted from peripheral blood using a method modified from Miller et al. (1988). This protocol consists of a salting-out method followed by a phenol-chloroform purification step. DNA extraction from saliva was carried out according to the manufacturer’s instructions (Oragene OG-250 kit; DNA Genotek, ON, Canada). Protocol: (http://www.dnagenotek.com/US/pdf/PD-PR-006.pdf)

Genetic analysis was conducted by Sanger sequencing for common variants in *CYP2D6*, *CYP2C9*, and *CYP2C19* genes. *CYP2D6* genotyping was conducted using a two-stage PCR. The first step used a previously described method (Wright et al., 2010) to isolate an amplicon approximately 6.6 kb in length. This step isolates a *CYP2D6* product from a neighboring *CYP2D7* pseudogene. This step also allows the identification of *CYP2D6* duplication or deletion alleles using specific primers. In brief, the initial PCR consisted of a 10 µl reaction which was set-up as follows: 1X KAPA LR reaction buffer (Kapa Biosystems, Wilmington, USA), 1.75 mM Mg^2+^, 0.3 mM of each deoxynucleoside triphosphate (dNTP), 0.4 µM of each 6.6 kb primer, 0.3 µM of duplication or deletion primers, 1 M betaine, 0.25 U of KAPA LR DNA (Kapa Biosystems, Wilmington, USA) polymerase, and 50 ng of DNA. Cycling conditions included initial heating to 94°C for 3 min, followed by 35 cycles of 94°C for 25 s, 68°C for 10 s, and 68°C for 7 min, and a final elongation step of 72°C for 7 min. Four µl of the initial PCR product was run on a 1% agarose gel to confirm the successful amplification of a 6.6 kb product and identification of any 3.5 kb duplication or deletion bands. Duplication or deletion alleles were confirmed by comparison with in-house controls (known samples with *CYP2D6* duplication or deletion). The second stage of the PCR utilized a 1,000 fold diluted PCR product (6.6 kb PCR product from first stage) to conduct a nested PCR to cover the *CYP2D6* gene. For the second stage, a standard 20 µl reaction was set-up as follows: 1X Fisher Biotec reaction buffer, 2.5 ng/µl template, 0.2 mM of each dNTP, 0.2 µM of each forward and reverse primer, 1.5 mM Mg^2+^, and 0.025 u/µl of TAQ-TI DNA polymerase (Fisher Biotec, Wembley, WA, Australia). This method allows for the identification of common alleles referred to as *2, *3, *4, *6, *7, *8, *9, *10, and *41, as well as other variants not often assessed in commonly available assay kits.

*CYP2C19* (*2–*8 and *17) and *CYP2C9* (*2, *3, *8) genotyping was conducted to assess common variants known to affect drug pharmacokinetics. A 20 µl PCR reaction was set-up as described above with *CYP2C19* and *CYP2C9* specific primers. Cycling conditions were as follows: initial heating to 94°C for 2 min, followed by 15 cycles of 94°C for 15 s, 65°C for 15 s (reducing by 1 degree per cycle), 72°C for 1 min. Followed by 20 cycles of 94°C for 15 s, 50°C for 15 s, and 72°C for 1 min.

After confirmation of PCR products on a 1% agarose gel, PCR products were prepared for bi-directional Sanger sequencing which was carried out on an Applied Biosystems 3130*xl* genetic analyzer for capillary electrophoresis. Bioinformatic analysis of generated sequences was done with the software Geneious V8.1.9 (Biomatters, Ltd., Auckland, New Zealand). In brief, generated sequencing files were aligned against the reference gene sequences from the National Center for Biotechnology Information (NCBI) (https://www.ncbi.nlm.nih.gov/gene) of *CYP2D6* (ID: 1565), *CYP2C19* (ID: 1557) and *CYP2C9* (ID: 1559). These reference gene sequences were annotated with variants from the Pharmacogene Variation (PharmVar) consortium (www.pharmvar.org) (Gaedigk et al., 2018). Participant samples were assigned “star” genotypes using translation tables available on PharmGKB. For example the *CYP2D6* translation table is available here (https://www.pharmgkb.org/gene/PA128/haplotype) (Whirl-Carrillo et al., 2012).

CYP2D6, CYP2C9, and CYP2C19 metabolizer status was assigned using CPIC (Clinical Pharmacogenetics Implementation Consortium) guidelines (Caudle et al., 2017).

### Psychometric Measures

The following measures were obtained using online or pen-and-paper self-report instruments:

K10: 10-item measure of past 1-month psychological distress (Kessler et al., 2003). Each item is scored from 1 to 5 giving a possible range of 10–50 on the whole instrument. Scores of 22 or more denote “high psychological distress” (Oakley-Browne et al., 2010)
Short Health Anxiety Inventory 18-item version (SHAI-18) (Salkovskis et al., 2002)
Personality traits: NEO-FFI 60-item version (McCrae and Costa, 2004)

### Bias

Sampling *via* social media is likely to identify potential participants who are active on social media platforms and interested in mental health topics. This convenient sampling strategy was chosen because we wanted to obtain a community sample of people who had taken an antidepressant and this was the most efficient way of doing so. Identifying patients *via* clinicians in primary or secondary care would have identified patients with more severe or unusual patterns of drug response, and possibly higher levels of distress, which was not the intended target population.

### Study Size

A power analysis was performed *a priori* and was used to guide recruitment. Assuming typical frequencies of uncommon CYP phenotypic variants being 5% in the control population, a total sample of 200 (125 cases and 75 controls) would give 80% power (with 5% type I error rate) to detect uncommon phenotypic variants which are present in at least 10% of cases. We considered this difference would be clinically significant if we were to detect it.

### Quantitative Variables and Statistical Methods

All analyses were conducted using IBM SPSS 25. Chi-square tests or independent sample t-tests were used for statistical comparisons between cases and controls. Fisher’s exact test was used to compare the frequency distribution of *CYP2D6*, *CYP2C19*, and *CYP2C9* phenotypes between cases and controls.

## Results

The final sample size fell slightly short of the *a priori* target (n = 200) because of dropout between the initial enrolment phase and genetic analysis. **Table 1** shows the characteristics of participants included in the case (n = 89) and control (n = 105) groups. Participants were predominantly female (87.1%) and they were aged 17 to 67 (mean 32.7). With respect to ethnicity, 70% of study participants identified as either European or New Zealand-European, 11% as Maori or Pacifica and the remainder consisted of Asian (4%) or unspecified ethnic groups. Current antidepressant use was reported by 96/105 (93.1%) controls and 68/89 (76.1%) cases.

**Table 1:** Participant demographics and Medication use.

	Cases (n = 89)	Controls (n = 105)	Total sample (n = 194)	Statistical test (p value for χ^2^ or t-test: cases *vs.* controls)
% Female	89.9	84.8	87.1	p = .57
Age: mean (sd)	30.0 (11.2)	35.0 (12.9)	32.7 (12.4)	p = .005

**Current antidepressant (n**; **%)**				
Fluoxetine	3 (3.4%)	20 (19.0%)	23 (11.3%)	
Citalopram or escitalopram	11 (14.3%)	32 (30.4%)	43 (22.2 %)	
Sertraline	15 (16.9%)	13 (13.7%)	28 (14.4%)	
Paroxetine	0 (0%)	3 (2.9%)	3 (1.5%)	
Venlafaxine	23 (25.8%)	23 (21.9%)	46 (23.7%)	
Other/unknown^1^	7 (7.9%)	2 (2.0%)	9 (4.6%)	
Tricyclic^2^	9 (9.0%)	2 (2.0%)	11 (5.7%)	
No antidepressant	21 (23.9%)	7 (6.9%)	28 (14.4%)	
Any antidepressant	68 (76.4%)	96 (91.4%)	164 (84.5%)	

**Current other drugs (n, %)**				
Mood stabilizer^3^	3 (3.4%)	9 (8.6%)	9 (4.6%)	.13
Antipsychotic^4^	12 (13.5%)	15 (14.3%)	27 (13.9%)	.87
Sedative/hypnotic^5^	24 (27.0%)	17 (16.2%)	43 (22.2%)	.07

^1^Mirtazapine (five cases, two controls), St John’s Wort (one case), bupropion (one case).

^2^Amitriptyline (three cases), nortriptyline (five cases, one control), clomipramine (one case).

^3^Lithium or valproate.

^4^Quetiapine (10 cases, 10 controls), olanzapine (0 cases, 2 controls), aripiprazole (1 case, 1 control), risperidone (1 case, 2 controls).

^5^Benzodiazepines, gabapentin, pregabalin or zopiclone.

Among the cases, 28/89 (31.4%) had not tolerated fluoxetine at some time in the past (including three *CYP2D6* PMs and one *CYP2C19* PM); 42/89 (47.2%) had not tolerated citalopram or escitalopram (including six *CYP2D6* PMs and one *CYP2C19* PM); and 14/89 (15.7%) had not tolerated sertraline (one *CYP2D6* PM, one *CYP2C19* PM, and one participant who was a PM for both genes). Among those who did not tolerate paroxetine (9/89; 10.1%) or venlafaxine (9/89; 10.1%) there were no *CYP2D6* or *CYP2C19* PMs. No participants reported exposure to fluvoxamine, desvenlafaxine, levomilnacipran, or duloxetine as these drugs are not available in New Zealand.

**Table 2** shows the distribution of *CYP2D6, CYP2C19*, and *CYP2C9* phenotypes [PM; IM; NM; RM (*CYP2C19* only) and UM] for cases and controls. There were no statistically significant differences in phenotype distribution between cases and controls for any of the *CYP* genes.

**Table 2:** CYP2D6, CYP2C9, and CYP2C19 phenotype for cases and controls.

	Case (n = 89)	Control (n = 105)	Statistical test
CYP2D6 phenotype: n (%)			
Poor metabolizer	9 (10.1)	6 (5.7)	Fisher’s exact test p = .26
Intermediate metabolizer	29 (32.6)	45 (42.9)	
Normal metabolizer	51 (57.3)	53 (50.5)	
Ultra-rapid metabolizer	0 (0)	1 (1.0)	

**CYP2C19: n (%)**			
Poor metabolizer	2 (2.2)	4 (3.8)	Fisher’s exact test p = .10
Intermediate metabolizer	29 (32.6)	20 (19.0)	
Normal metabolizer	32 (36)	43 (41)	
Rapid metabolizer	25 (28.1)	31 (29.5)	
Ultra-rapid metabolizer	1 (1.1)	7 (6.7)	

**CYP2C9 phenotype: n (%)**			
Poor metabolizer	1 (1.1)	6 (5.7)	Fisher’s exact test p = .24
Intermediate metabolizer	26 (29.2)	27 (25.7)	
Normal metabolizer	62 (69.7)	72 (68.6)	

Phenotypes are derived from (Caudle et al., 2017).

Cases and controls also did not differ significantly on NEO-FFI-3 personality dimensions or SHAI scores (**Table 3**). High levels of psychological distress were present across the whole sample, with 70.3% scoring 22 or more (denoting high distress) and 39.5% scoring 30 or more (denoting very high distress) (Oakley-Browne et al., 2010).

**Table 3:** Psychometric measures.

Psychometric measures				
	Cases (n = 89)	Controls (n = 105)	Total sample (n = 194)	Statistical test (p value for χ^2^ or t-test: cases *vs.* controls)
Short Health Anxiety Inventory (SHAI) score (SD)	16.2 (6.1)	15.2 (6.8)	15.7 (6.5)	.31
Psychological distress (K10) (SD)^1^	27.7 (8.0)	25.5 (8.3)	26.5 (8.2)	.07
Percentage with high psychological distress (K10≥22) (SD)	77.1%	65.0%	70.8%	.13

**NEO-FFI scores (SD)**				
Neuroticism	34.8 (7.1)	33.2 (8.8)	33.9 (8.1)	.18
Extraversion	22.0 (7.1)	22.3 (7.9)	22.2 (7.6)	.76
Openness to experience	33.0 (6.8)	33.2 (7.4)	33.1 (7.1)	.93
Agreeableness	34.3 (6.5)	33.8 (5.7)	34.0 (6.0)	.58
Conscientiousness	27.2 (9.0)	28.9 (8.7)	28.0 (8.9)	.19

^1^The version of the K10 which was used scores individual items from 1 to 5, therefore the possible total score ranges from 10 to 50. Standard deviation (SD).

**Table 4** presents case series data from participants identified as PMs for *CYP2D6* (n = 15), *CYP2C19* (n = 6), or CYP2C9 (n = 7) with one participant being a PM for both *CYP2D6* and *CYP2C19*. Notably, 7/15 *CYP2D6* PMs reported tolerability problems with drugs for which CYP2D6 is not the major pathway for metabolism (escitalopram, citalopram, or sertraline) while three reported tolerating moderate to high doses of paroxetine or fluoxetine, which are affected by CYP2D6. Similarly, while *CYP2C19* PMs were uncommon (n = 6), one *CYP2C19* PM tolerated escitalopram (metabolized to a large extent by CYP2C19) but not fluoxetine (minimally affected by CYP2C19). Two *CYP2C19* PMs were currently taking a drug metabolized by CYP2C19, but one of these participants had reduced the dose of sertraline from 150 to 100 mg in response to serotonergic side effects.

**Table 4:** Clinical histories of participants identified as CYP2D6, CYP2C9, or CYP2C19 poor metabolizers.

Case or control	SSRI or SNRI (s) not tolerated	*CYP2D6* genotype (phenotype)	*CYP2C19* genotype (phenotype)	*CYP2C9* genotype (phenotype)	Current daily antidepressant dose	Outcome of past antidepressant therapy
**CYP2D6 poor metabolizers**						
Case	Fluoxetine, escitalopram	*4/*4 (PM)	*1/*1 (NM)	*1/*1 (NM)	Venlafaxine 225 mg	Fluoxetine: night sweats, vivid nightmares, and difficulty regulating temperature. Increased anxiety in first few weeks of treatment
Case	Escitalopram, sertraline	*4/*6 (PM)	*1/*1 (NM)	*1/*2 (IM)	Venlafaxine 150 mg	Lethargy, lack of motivation, and suicidal thinking with sertraline. Lethargy with escitalopram
Control		*4/*4 (PM)	*1/*1 (NM)	*1/*2 (IM)	Citalopram 20 mg	No difficulty tolerating citalopram
Case	Escitalopram	*4/*4 (PM)	*1/*17 (RM)	*1/*2 (IM)	None	Ceased escitalopram due to unspecified side effects
Control^a^		*4/*4 (PM)	*2/*2 (PM)	*1/*1 (NM)	Sertraline 100 mg	Sertraline 150 mg per day: insomnia, sweating, tremor, and increased anxiety. Dose reduced to 100 mg per day
Control		*4/*4 (PM)	*1/*1 (NM)	*1/*2 (IM)	Citalopram 20 mg	No difficulty tolerating citalopram
Case	Citalopram	*4/*4 (PM)	*1/*2 (IM)	*1/*1 (NM)	Sertraline 50 mg	Citalopram: nausea, constipation, and dysmenorrhea
Control		*4/*4 (PM)	*1/*1 (NM)	*1/*1 (NM)	Citalopram 40 mg	No difficulty tolerating citalopram
Case	Sertraline	*3/*4 (PM)	*1/*2 (IM)	*1/*1 (NM)	Sertraline 100 mg	Sertraline 100 mg per day: night sweats. Trialed a reduction to sertraline 50 mg before increasing dose back to 100 mg
Control		*4/*4 (PM)	*1/*1 (NM)	*1/*2 (IM)	Venlafaxine 300 mg	Citalopram, escitalopram, fluoxetine, and sertraline had all been trialed and stopped due to lack of efficacy rather than side effects
Control		*4/*4 (PM)	*1/*2 (IM)	*1/*2 (IM)	Venlafaxine 300 mg	Previously tolerated paroxetine 40 mg per day. Venlafaxine 300 mg per day: reduced appetite but otherwise tolerated.
Case	Fluoxetine	*4/*4 (PM)	*1/*17 (RM)	*1/*1 (NM)	Citalopram 20 mg	Fluoxetine: “extreme sleepiness.” Tolerated citalopram
Case	Citalopram	*4/*4 (PM)	*1/*17 (RM)	*1/*2 (IM)	Nil	Citalopram: QTc prolongation. Tolerated fluoxetine 60 mg per day
Case	Escitalopram	*4/*4 (PM)	*1/*1 (NM)	*1/*2 (IM)	Nil	Escitalopram: neck spasms, weight loss, fatigue, dry mouth, anxiety, suicidal thoughts, and hallucinations
Case	Fluoxetine, citalopram	*4/*4 (PM)	*1/*17 (RM)	*1/*1 (NM)	Venlafaxine 150	Fluoxetine: nausea and “heartburn.” Citalopram: dizziness, concentration difficulties, sedation, and increased suicidality
**CYP2C19 poor metabolizers**						
Case	Sertraline	*1/*1 (NM)	*2/*2 (PM)	*1/*1 (NM)	Nil	Sertraline: nausea and diarrhea. Tolerated citalopram
Control	Citalopram	*1/*2 (NM)	*2/*2 (PM)	*1/*1 (NM)	Nil	Citalopram: increased suicidality but no side effects.
Control	Sertraline	*4/*4 (PM)	*2/*2 (PM)	*1/*1 (NM)	Sertraline 100 mg	Sertraline 150 mg per day: insomnia, sweating, tremor, and increased anxiety. Dose reduced to 100 mg per day.
Case	Fluoxetine	*1/*1 (NM)	*2/*4 (PM)	*1/*2 (IM)	Escitalopram 20 mg	Fluoxetine: excessive sweating. Escitalopram well tolerated
Control		*2/*3 (IM)	*2/*2 (PM)	*1/*1 (NM)	Venlafaxine 225 mg + mirtazapine 15 mg + amitriptyline 10 mg	Previously prescribed citalopram and paroxetine. Stopped due to lack of efficacy rather than side effects
Control		*1/*2 (NM)	*2/*2 (PM)	*1/*1 (NM)	Fluoxetine 20 mg	No difficulty tolerating fluoxetine 20 mg daily
**CYP2C9 poor metabolisers**						
Control		*1/*1 (NM)	*1/*1 (NM)	*3/*3 (PM)	Paroxetine 20 mg + amitriptyline 35 mg	Tolerating paroxetine 20 mg daily
Control		*2/*41 (NM)	*1/*1 (NM)	*3/*3 (PM)	Citalopram 20 mg	Previously tolerated fluoxetine 20 mg daily but stopped due to lack of efficacy. No side effects on citalopram
Control		*1/*2 (NM)	*1/*1 (NM)	*2/*2 (PM)	Citalopram 20 mg	No difficulty tolerating citalopram
Control		*1/*4 (IM)	*1/*1 (NM)	*2/*3 (PM)	Fluoxetine 20 mg daily	No difficulty tolerating fluoxetine
Case	Sertraline, fluoxetine	*1/*1 (NM)	*1/*1 (NM)	*2/*3 (PM)	Amitriptyline 20 mg daily	Sertraline 100 mg daily was associated with “fatigue and brain fog.” Fluoxetine was also associated with similar symptoms.
Control		*1/*4 (IM)	*1/*1 (NM)	*2/*2 (PM)	Venlafaxine 375 mg + nortriptyline 12.5 mg	Previously tolerated citalopram 80 mg daily apart from some sexual dysfunction. Switched to venlafaxine due to lack of efficacy not side effects
Control		*1/*4 (IM)	*1/*1 (NM)	*2/*3 (PM)	Amitriptyline 20 mg daily	Previously tolerated citalopram and venlafaxine

NM, normal metabolizer; IM, intermediate metabolizer; PM, poor metabolizer; RM, rapid metabolizer; UM, ultra-rapid metabolizer. Phenotype in () is derived from (Caudle et al., 2017).

a This participant was a poor metabolizer for both CYP2D6 and CYP2C19. Participant was categorized as a control despite dose-dependent side effects, because she had not reduced the dose by at least 50% in response to side effects, as required by the a priori case definition.

No *CYP2D6* PMs reported currently taking a drug whose metabolism was substantially affected by CYP2D6: their current antidepressant therapy was citalopram (n = 4), sertraline (n = 3), venlafaxine (n = 5), no antidepressant (n = 2), and in one participant, it was unclear.

## Discussion

The main premise of personalized antidepressant prescribing is that it helps to improve drug tolerability and avoid toxicity. While improved remission rates have been reported using genetic decision support algorithms (Singh, 2015), this finding has not yet been reliably replicated and the area remains somewhat controversial (Bousman and Hopwood, 2016; Singh and Bousman, 2017; Bousman and Muller, 2018). The clinical utility of such decision support tools is predicated on the belief that most antidepressant intolerability is linked to known genetic variants in pharmacogenes, which can be easily and cheaply detected. However, if antidepressant tolerability is not strongly related to genetic variability, such decision support tools may do little to improve antidepressant tolerability in a pre-emptive testing scenario. They may therefore be more valuable as an *ad hoc* tool in selected high-risk clinical samples (Nassan et al., 2016). Furthermore, while the premise of the technology is that it improves clinical outcomes and therefore lowers costs, it increases the complexity of clinical care. The technology might also increase inequality if it is only available in high-income countries or to more privileged or educated patient groups. Direct-to-consumer genetic testing raises even more complex ethical issues, and it has been argued it should be accompanied by genetic counseling (Bousman and Hopwood, 2016; Bousman et al., 2019).

We observed a low correlation between phenotypes and self-reported adverse outcomes among *CYP2D6* and *CYP2C19* PMs identified in our cohort. For example 8 of the 15 *CYP2D6* PMs had experienced tolerability problems with an antidepressant *not* metabolized CYP2D6, i.e., citalopram or sertraline (Hicks et al., 2015). Conversely, several *CYP2D6* PMs had no apparent trouble tolerating moderate to high doses of antidepressants for which *CYP2D6* PM status is thought to induce side-effects, and for which CPIC guidelines recommend an alternative antidepressant (Hicks et al., 2015). The findings for *CYP2C19* PMs were similarly inconsistent, albeit that their smaller numbers makes them harder to interpret. With respect to *CYP2C19* and citalopram/escitalopram, a recent meta-analysis of over 4,000 patients by Fabbri et al. (2018) reported that compared to normal metabolizers, *CYP2C19* PMs had a higher risk of gastrointestinal, neurological, and sexual side effects. However, *CYP2C19* PMs had significantly increased rates of remission as well as marked improvement in symptoms of depression when compared with normal metabolizers (Fabbri et al., 2018).

Prior research indicates that the SNPs we investigated in *CYP2D6* and *CYP2C19* genes are associated with significant changes in plasma levels of SSRIs and SNRIs (Preskorn, 2003a; Preskorn, 2003; Gardiner and Begg, 2006; Shams et al., 2006). However, consistent with our findings, some previous studies found these SNPs are not predictive of side effects or antidepressant efficacy (Hodgson et al., 2015; Taranu et al., 2017). In addition, Serretti et al. (2009) showed no association between pharmacogenes (2D6, 2C19, 2C9, and 1A2) and response or remission to antidepressants (Serretti et al., 2009). The lack of an apparent signal in our study could also be explained by a “nocebo effect”(18) from antidepressants which overshadowed more specific dose-dependent side effects. Nocebo effects involve non-specific side effects which patients report after a treatment starts. They are analogous to the non-specific benefits which are often labeled as placebo effects. Although nocebo effects have been less well studied than placebo effects, the phenomenon appears more common in women and those with psychological symptoms (Wells and Kaptchuk, 2012), who made up the majority of our sample.

Despite most participants in this study currently being prescribed an antidepressant or having recently taken one, over two thirds were still reporting high psychological distress. While it is possible that people with a poor response to antidepressant treatment were more likely to take part in the study, it does speak to the ineffectiveness of these drugs for many people. Contrary to our *a priori* hypothesis, psychological distress, health anxiety, and trait anxiety (neuroticism) were not higher in cases compared to controls suggesting these psychological factors did not explain why some participants had not tolerated antidepressants. This is surprising since psychological factors are thought to affect drug tolerability in general (Barsky et al., 2002). Furthermore, the mean level of health anxiety among cases was well below the levels previously reported in patients with hypochondriasis (Alberts et al., 2013). It is possible the high current distress levels across the whole sample created a “ceiling effect” which obscured between-group differences in anxiety. Alternatively, prescribers are often skilled at detecting anxiety-prone patients: the lack of difference in state and trait anxiety measures between cases and controls might therefore simply reflect prescribing practices to avoid drug intolerability in patients whose expectations made them at risk of side effects.

The limitations of this study include the use of a non-random sampling strategy, which resulted in a low proportion of male participants in particular. Caution is therefore needed in generalizing the findings to a wider population of people who have taken antidepressants. The heterogeneity of the drug exposure history in the sample was a further limitation: since patients had failed to tolerate a range of SSRIs and SNRIs, each with different CYP metabolism, it is possible this heterogeneity overshadowed any signal for individual drugs. Due to budgetary constraints, our study did not assess antidepressant or metabolite levels in participants. Future studies assessing antidepressant adverse drug reactions (ADRs) or efficacy should consider this during the design phase.

An initial power analysis conducted assuming typical frequencies of uncommon CYP gene variants being 5% in the control population suggested that a sample of 125 cases would give 80% power (with 5% type I error rate) to detect a gene variant which is present in 10% of cases, a difference we considered would be clinically significant. However, our recruitment of cases was not focused on a specific drug-CYP-ADR, which needs to be considered in the future. Alternatively, a much larger sample might have allowed more robust subgroup analyses according to the drug(s) that were not tolerated and also allowed for analysis of phenoconversion by analysis of concomitant medications. Information about drug exposure was taken from self-report, and while most participants’ reports were likely to have been accurate, this was not verified against medical records except in cases where data were unclear. We have previously reported (Maggo et al., 2019) a high burden of pharmacogenetic variation in common *CYP450* genes in patients who reported ADRs to commonly prescribed psychiatric medication. The major difference being, this small cohort of patients was selected by a specialist mental health pharmacist, and not self-referred as in the current study. Finally, we elected to use a clear behavioral phenotype (antidepressant reduction or discontinuation) rather than a structured adverse effect reporting tool, but this might have led to under-recognition of a subset of adverse effects which were more specific indicators of drug toxicity, for example serotonin toxicity symptoms.

Several clinical trials on genetically informed antidepressant prescribing have been either recently completed or are still recruiting (see www.clinicaltrials.gov). As the findings from these studies become available, the role of pharmacogenetic testing to guide antidepressant therapy will become clearer. Our findings suggest that most patients who self-report difficulty tolerating SSRI or SNRI therapy recruited through social media do not have a clear pharmacogenetic explanation for this. However, as already discussed, sample-size and methodological limitations do not allow strong conclusions to be made from the present study.

## Data Availability Statement

The datasets for this manuscript are not publicly available because they may contain identifying participant information. Anonymised data such as phenotype/genotype and case/control classification can be requested. Requests to access the datasets should include a letter indicating the intended use and appropriate approval by your institution. This should be directed to the corresponding author.

## Ethics Statement

The studies involving human participants were reviewed and approved by The New Zealand Northern B Health and Disability Ethics Committee. The patients/participants provided their written informed consent to participate in this study. Written informed consent was obtained from the individual(s) for the publication of any potentially identifiable images or data included in this article.

## Author Contributions

SM: Recruited and consented the 194 study participants, collated psychometric and personal information, conducted genetic testing, assigned phenotypes and assisted in the write-up of the manuscript. MK: Provided advice on pharmacogenetic aspects of this manuscript. ZB: Assisted in the collation of the NEO-FFI assessment. AM: Assisted in the extraction of DNA and genetic testing of samples. JR: Assisted with social media advertisement and recruitment of study participants. RM: Provided professional unbiased input on assignment of participants as cases or control. JF: Principal investigator and holder of the research grant produced the first draft of this manuscript, conducted all statistical testing and collation of results into tables.

## Funding

This study was funded by a University of Otago Research Grant. ZB’s salary was funded by a Summer Studentship jointly funded through University of Canterbury Foundation and Canterbury Medical Research Foundation. SM’s salary was partly funded by the Jim and Mary Carney Charitable Trust (Whangarei, New Zealand).

## Conflict of Interest

The authors declare that the research was conducted in the absence of any commercial or financial relationships that could be construed as a potential conflict of interest.
